# A qualitative evidence synthesis of the experiences and perspectives of communicating using augmentative and alternative communication (AAC)

**DOI:** 10.1080/17483107.2022.2105961

**Published:** 2022-08-26

**Authors:** Katherine Broomfield, Deborah Harrop, Georgina L. Jones, Karen Sage, Simon Judge

**Affiliations:** a Department of Adult Speech and Language Therapy, Gloucestershire Health and Care NHS Foundation Trust, Gloucestershire, UK; b Department of Nursing, Manchester Metropolitan University, Manchester, UK; c Centre for Health and Social Care, Sheffield Hallam University, Sheffield, UK; d Department of Psychology, Leeds Beckett University, Leeds, UK; e Barnsley Assistive Technology Team, Barnsley Hospitals NHS Foundation Trust, Barnsley, UK; f School of Health and Related Research, University of Sheffield, Sheffield, UK

**Keywords:** Augmentative and alternative communication, qualitative evidence synthesis, experiences, perspectives, patient-reported outcome measure

## Abstract

**Purpose:**

This paper presents a review of the existing qualitative research literature concerning people’s experience of communicating using augmentative and alternative communication (AAC). The aims of conducting this review were to find out more about the values and outcomes that are important to people about the AAC they use to support their communication. This review was conducted to provide a deeper understanding of these experiences to inform the development of a patient-reported outcome measure (PROM).

**Materials and methods:**

A qualitative evidence synthesis of existing qualitative research literature was undertaken to explore and appraise current knowledge about the experiences of people who use AAC.

**Results:**

From 115 qualitative research reports 19 papers were identified that responded directly to the research question and aims of the review. Data were identified that could be organized within an a priori framework consisting of the constructs of values, outcomes, and context.

**Conclusion:**

The review has resulted in a deeper, analytical understanding of the experiences of people who require AAC. The results indicate a set of concepts that can be used to inform the development of a PROM. A PROM can be used to assist clinicians and researchers to better understand the perspectives of people who require AAC and evaluate interventions. The results also encourage professionals to reconsider the terminology and methods used when working alongside people who require AAC and to reflect on the multidimensional factors that influence people’s experience of communication.

## IMPLICATIONS FOR REHABILITATION

- Patient-reported outcome measures (PROMs) can be useful tools to support clinician-patient communication, facilitate shared decision making and establish priorities for rehabilitation.
- It can be difficult to engage people who have complex communication difficulties in decisions about the important outcomes to them from using augmentative and alternative communication (AAC).
- This qualitative evidence synthesis provides a deeper understanding of the experiences and perspectives of people who use AAC.
- The results will be used to inform the development of a PROM which can be used to facilitate shared decision-making, and evaluate AAC interventions from the perspective of the people who use these technologies.

## Introduction

Augmentative and alternative communication (AAC) refers to a set of tools use by people who cannot express themselves clearly using their natural speech alone. These tools consist of unaided options such as gestures, signs and facial expressions and aided AAC, for example external paper-based systems and electronic or computer-based devices that generate synthesized speech output from messages either inputted or stored within them [1]. People who can benefit from AAC include children and young people who have communication difficulties from birth, and adults who have persistent communication difficulties from childhood or who acquire communication difficulties because of a medical condition. AAC devices are frequently provided and supported by healthcare professionals and it is these aided systems that are the focus of the current research. It is important for clinicians working with people who use AAC to understand the value or meaning ascribed to AAC in order to be able to support them to use an AAC device effectively [2]. The perceived meaning and value of AAC can impact a person’s engagement with and therefore the overall utility of an AAC device [3]. Understanding the value placed on AAC involves open discussion, close collaboration, and shared decision making between the individual and the clinician supporting them. When working with those who will use AAC it is the responsibility of clinicians to establish goals, targets, or outcomes in order that they can plan appropriate provision, support, and training. There is currently little agreement about what are important outcomes of AAC from the perspective of the person who require AAC [4,5]. People who have communication difficulties and may benefit from AAC may find it difficult to engage in discussions about their values and/or set goals from which to evaluate outcomes from AAC [6] and are rarely involved in decision making about AAC [7].

Patient-reported outcome measures (PROMs) can be useful clinical tools to support clinician-patient communication [8] and so can be used to establish significant values and important outcomes from the patient perspective. The use of a patient-reported outcome measure (PROM) is not credible unless there is evidence that it has been validated with the population of interest [9]. There are tools that have been developed to understand the attitudes of young people towards AAC [10] and to measure the impact of AAC on families [11] but there are currently no PROMs validated to measure the outcomes of AAC from the perspective of those who use AAC devices [12]. A PROM should measure a specific concept or set of concepts, known as a conceptual framework, that has been developed with the intended population [9] in order that the outcomes of importance are evaluated. Existing frameworks and theories within the literature which could inform a conceptual framework describe AAC in terms that broadly relate to functional outcomes such as communication competence [13], or focus on more specific skill development outcomes such as operational use of AAC and linguistic ability. These frameworks have not been developed in conjunction with people who use AAC nor is it clear whether they reflect the outcomes or values of importance to this population [14].

Potential items which might be included in a PROM can be developed by bringing together relevant concepts, ideas, and parameters from existing literature with the thoughts and experiences of those who have an expert opinion [9]. There is an inherent difficulty with collecting rich qualitative data from the population who use AAC [15] and a tendency for data analyses to be descriptive rather than analytic [16–18]. Themes generated in qualitative studies conducted with people who use AAC tend to be presented in terms of barriers and facilitators to use rather than demonstrating a deeper critique of the experiences of communicating using AAC [19]. Research into people’s perspectives of AAC also often: (a) relies on reports from family members or proxies [11,16]; (b) focuses on specific AAC devices such as speech generating devices (SGDs) or Talking Mats [20,21]; or (c) has explored the experiences of people who use AAC with regards to specific occupations or activities, for example mentoring, accessing leisure, or volunteering [20,22,23]. Identifying the outcomes that are a priority for people who use AAC involves understanding the nature of their experience regardless of the type of device that they use or the condition that underlies their communication impairment.

Qualitative research enquiry is a set of methods aimed at developing an understanding of the essence of experience and the range of opinion concerning a specific topic [24] and is frequently used to inform PROM development. Qualitative evidence synthesis (QES) is the method by which targeted qualitative research literature is systematically appraised to look beyond the individual analyses and find commonalities across, between, and within the identified papers [25]. The aim of the qualitative evidence synthesis reported here is to identify, review, and appraise existing research literature concerning people’s experiences of communicating using AAC to understand what is important and of value to them, with particular focus on the outcomes of using AAC as perceived by those who use AAC devices.

The research question for this review was: What are the experiences of communicating using AAC as reported by people who use AAC devices? To address this question, two review aims were identified:

1. What are the values of people who use AAC with regards to communication?
2. What outcomes are important to people who use AAC?

## Method

### Participants

To address the specific research question, inclusion/exclusion criteria were drawn up that would identify studies that reported the perspectives of people who use AAC rather than family members, professionals, or proxies. Studies were included in the QES if the derived data were from: (a) a population who had used AAC, (b) participants 12 years and over, (c) there were identifiable data that came directly from participants who use AAC, (d) the data concerned the experience of communicating using AAC devices (including in-person and online communication, *via* social media).

Studies were excluded based on the following criteria: (a) participants were reported as having a severe intellectual disability or significant cognitive impairment affecting reasoning and judgement, (b) participants who exclusively used manual signs, gesture, facial expression, or non-verbal communication, (c) participants with autism spectrum conditions (ASC) or social communication difficulties, (d) brain-computer interface papers, (e) speech recognition literature, (f) papers exclusively concerned with assistive devices for hearing or visual impairment, (g) proxy reports of experience, (h) reviews and opinion pieces, (i) literature reporting broader experiences of AAC users (e.g., leisure, work, relationships), (j) foreign language papers where no translation was available, and (k) grey literature such as non peer-reviewed reports, magazines and blogs.

### Research design

The QES was conducted following the method for conducting evidence syntheses described in guidance produced by Popay et al. [26] and is reported using the ENTRQ (enhancing transparency in reporting the synthesis of qualitative research) framework [27]. In brief, the guidance stipulates that reviews should be (a) based on a specific review question, (b) have a systematic process to identify studies to include using defined a search strategy and screening procedure, (c) complete a study quality appraisal, (d) extract and synthesize the data, and (e) disseminate the findings. A protocol for the review was written and published on PROSPERO (https://www.crd.york.ac.uk/PROSPERO/display_record.php?RecordID=120121).

#### Patient and public involvement

Patient and public involvement (PPI) in research is described as “research ‘with’ or ‘by’ members of the public rather than ‘to’, ‘about’ or ‘for’ them” [28, p. 6]. In the United Kingdom (UK), PPI is central to ensuring the relevance, accountability, and impact of health research. A PPI group consisting of seven people who use AAC, as well as their family members and carers, supported this QES by:

1. Contributing to developing the inclusion/exclusion criteria;
2. Providing advice to the research team during screening of papers for the QES;
3. Designing a framework with the research team to support the descriptive analysis of data;
4. Supporting dissemination of results by reviewing the final report and associated presentations.

Further details about the people within the group and how they contributed to the wider research study has been written and published elsewhere [29].

### Procedures

#### Strategy

To initiate the QES, a broad search strategy was adopted incorporating population terms adapted from previous systematic reviews relating to people who use AAC [4,12] and qualitative research terms [30]. Mixed methods papers as well as qualitative papers were included in the QES where qualitative data could be disambiguated and extracted. Search terms were entered into Medline (EBSCO), CINAHL (EBSCO), PsycINFO (ProQuest), ERIC (EBSCO) and Scopus (Elsevier) from inception to January 2019. All papers containing qualitative data pertaining to the research questions were included. The search strategy is available on PROSPERO.

#### Screening

Papers were sifted for inclusion in the QES by title and abstract by one author (KB), 10% were checked by a second member of the team (DH). One author (KB) read the full-text papers identified by the sifting process and a second author (SJ) checked 10% of the full text papers. Consensus on papers to include in the QES was achieved through discussion in relation to the study protocol and inclusion/exclusion criteria at both stages of screening.

#### Quality appraisal

The Specialist Unit for Review Evidence (SURE) [31] tool was used to evaluate the quality of reporting in the research papers as it provides a descriptive summary of particular aspects of the research report and highlights specific areas of reporting strength and limitation. Quality appraisal was carried out by one author KB. KB weighted and numerically ranked the papers to provide a grading to the papers based on how completely they fulfilled the parameters set out in the SURE checklist. This ranking mechanism informed the data extraction process (described in further detail below) and the final confidence assessment as the team were able to check that data from high quality reports were strongly represented in the final analytic synthesis. The final analytic synthesis of the data was subject to an overall Confidence in Evidence Reviews of Qualitative research (CERQual) assessment, also carried out by KB then discussed with the wider team. CERQual involves assessing findings based on their methodological limitations, the coherence of the finding, the adequacy of the data supporting a finding, and the relevance of the data from the primary studies to the finding. The final CERQual statement is a judgement about the overall confidence with which review findings can be assessed based on concerns identified in relation to these four areas [32].

#### Data extraction and synthesis

Data about the paper describing the study, population, and methods were extracted into a table specifically designed for the purposes of this review (Table 1). The results sections of each paper were then extracted into NVivo^TM^ 11 (NVivo for Mac, version 11.4.3). Thomas and Harden’s [25] procedure for thematic synthesis was employed to appraise the data derived from the results sections of the included papers and involved: (a) line-by-line coding, (b) development of descriptive themes, and (c) development of analytic themes. Starting with the highest quality papers, as they were ranked following SURE quality appraisal and weighting process, extracted data were coded line-by-line and collected into descriptive themes using Nvivo^TM^ 11 by KB. These descriptive themes were presented to the patient and public involvement (PPI) group for discussion and triangulation to check that they resonated with the experiences of group members [25]. Two *a priori* overarching constructs, defined by the aims of the QES and with a view to inform the development of a conceptual framework for a PROM (values and outcomes), were also presented to the group with the intention that these were to inform further analytic appraisal of the results. A third construct, context, was added to the *a priori* framework following the discussion with PPI group about the descriptive themes.

**Table 1.** Description of studies included in the review.

Study details			Participant details				Methodology details			
Authors	Title	Country	Underlying medical condition	Age	Sample size (–)	Gender	Study design	Aims	Data collection	Analyses
Bay [37]	Communicating through social media: how persons with ALS use the internet to maintain social connections	USA	Amyotrophic lateral sclerosis (ALS)	49–73	*n* = 8	5 female, 3 male	Parallel mixed methods	To explore the social participation experiences and perspectives of pALS [sic] who use AAC to inform current models of practice	Questionnaire, web-based interviews and video-recordings	Inductive analysis and constant comparison
Bruce, et al. [48]	Writing with voice: an investingation of the use of a voice recognition system as a writing aid for a man with aphasia	UK	Stroke	57	*n* = 1	1 male	Case study	Investigation whether a man with fluent aphasia could learn to use Dragon Naturally Speaking to write.	Email feedback	Descriptive
Caron & Light [43]	“My world has expanded even though I'm stuck at home”: Experiences of individuals with amyotrophic lateral sclerosis who use augmentative and alternative communication and social media.	USA	Amyotrophic lateral sclerosis	35–76	*n* = 9	4 female, 5 male	Qualitative: focus group	This study aimed to expand the current understanding of how persons with amyotrophic lateral sclerosis (pALS) use augmentative and alternative communication and social media to address their communication needs.	Online focus group	Thematic analysis
Caron & Light [47]	Social media experiences of adolescents and young adults with cerebral palsy who use augmentative and alternative communication.	USA	Cerebral palsy	14–21	*n* = 7	4 female, 3 male	Qualitative pilot study: Focus group	To expand the current understanding of how adolescents and young adults with cerebral palsy (CP) and complex communication needs use social media.	Online focus group	Thematic analysis
Carpe, et al. [35]	Perceptions of writing and communication aid use among children with a physical disability	Canada	Cerebral palsy, spinal tumour	8–18	*n* = 8	5 females, 3 males	Qualitative	To explore the perceptions of children with physical disabilities regarding their writing and communication aids.	In-depth, semi-structured interviews and focus group	Constant compar-ison: inductive approach to thematic analysis
Childes, et al. [38]	The use of technology for phone and face to face communication after total laryngectomy	Canada	Laryngectomy	64 (mean)	*n* = 17	6 female, 11 male	Mixed methods	To investigate the ways in which individuals who have undergone laryngectomy might use SGDs or other types of communication technology in place of verbal communication.	Online questionnaire	Descriptive
Chung, et al. [49]	Perspectives of high tech augmentative and alternative communication users with cerebral palsy at the post- secondary level	USA	Cerebral palsy	Early 30 s- mid 40 s	*n* = 5	2 female, 3 male	Qualitative case studies	To provide a better understanding of the communication challenges experienceed and the strategies used to face the harsh demands during transition years	In-depth interviews and observations	Not specified
Davis [17]	Reflections of nine participants regarding their experiences of being african-american and using augmentative and alternative communication across their lifespan at home, school, vocation, and community	USA	Cerebral palsy	26–54	*n* = 9	2 female, 7 male	Qualitative: Phenomenological	To describe and develop an understanding about the experience of being Non-European American (i.e. diverse) and using AAC	Semi-structured interviews: phenomenology	Phenomenology and thematic
Dickerson, et al. [33]	The meaning of communication: Experience with augmentative communication devices	USA	Cerebral palsy, traumatic brain injury, stroke, other neurological condition	12–69	*n* = 16	4 female, 12 male	Secondary analysis of narratives from a previous study	To increase our understanding of the importance of communication made possible by the [AAC] device	Secondary hermeneutic analysis	Phenomenological interpretation of narratives
Hodge [18]	Why is the potential of augmentative and alternative communication not being realized? Exploring the experiences of people who use communication aids	UK	Not stated	Adults	*n* = 19	2 female, 17 male	Qualitative	To develop an understanding of how the people who borrowed [AAC] equipment experience using that equipment	Semi-structured interviews	Not specified
Howery [36]	Out of time: The Experience of Speech generating device users	Canada	Cerebral palsy, Rett syndrome, degenerative neurological condition	Adolescents	*n* = 9	9 (gender not stated)	Qualitative	To provide new under- standings and insights into the meaning of phenomena …speaking with a SGD [speech-generating device]	Phenomenological investigation	Phenomenological enquiry
Iacono, et al. [41]	Experiences of adults with complex communication needs receiving and using low tech AAC: an Australian context	Australia	Developmental disability, traumatic brain injury	21–74	*n* = 15	6 female, 9 male	Qualitative	To explore how people who received low tech aids […] used them	Interviews	Content and thematic analysis
Johansson, et al. [44]	Communication difficulties and the use of communication strategies: from the perspective of individuals with aphasia	Sweden	Stroke	48–79	*n* = 11	4 female, 7 male	Qualitative	To explore how people with aphasia experience having conversations, how they handle communication difficulties, and how they perceive their own and their communication partners’ use of communication strategies	Semi-structured interviews; phenomenology	Content analysis
Lund & Light [46]	Long-term outcomes for individuals who use augmentative and alternative communication: Part III – contributing factors	USA	Cerebral palsy	19–23	*n* = 7	7 male	Qualitative	What factors contributed either positively or negatively to outcomes for a group of young men with complex communication needs?	Interviews	Thematic analysis
Martin, & Newell [42]	Living through a computer voice: A personal account	UK	Cerebral Palsy	Adult	*n* = 1	1 male	Personal experience report	To explore everyday practical issues and broader imaginative, philosophical and sociological implications of AAC technology	Interview	No analysis
Murphy [45]	“I prefer contact this close”: Perceptions of AAC by people with motor neurone disease and their communication partners	UK	Amyotrophic lateral sclerosis (ALS)/ Motor Neurone Disease (MND)	45–78	*n* = 15	7 female, 8 male	Qualitative case study	What is the purpose of the communication and the use of augmentative and alternative communication (AAC) according to the perceptions of people with MND and their partners were examined?	Semi-structured interviews	Cognitive mapping
Paterson & Carpenter [34]	Using different methods to communicate: How adults with severe acquired communication difficulties make decisions about the communication methods they use and how they experience them	UK	Stroke, amyotrophic lateral sclerosis, traumatic brain injury, Huntington’s disease	45–70	*n* = 7	7 male	Qualitative: Phenomenology	To explore how adults with severe acquired communication difficulties experience and make decisions about the communication methods they use.	Semi-structured interviews; phenomenology	Colaizzi’s phenomenological analysis framework
Smith & Connolly [40]	Roles of aided communication: perspectives of adults who use AAC	Ireland	Cerebral palsy	Adults	*n* = 18	Not stated	Mixed methods	To describe the supports available to adults using aided communication, their views on the role(s) they assigned to aided communication within their total communication systems and the factors they identified as affecting their use of aided communication	Structured questionnaire-based interviews	Descriptive
Trembath, et al. [23]	“Communication is Everything:” The Experiences of Volunteers who use AAC	Australia	Not stated	20–60	*n* = 24	12 female, 12 male	Qualitative	To explore the impact that using augmentative and alternative communication (AAC) had on the experiences of 24 adults with lifelong disabilities who worked as volunteers.	In-depth interviews and grounded theory	Grounded theory

During the analytic synthesis, themed data were organized by KB and SJ into the *a priori* constructs: values, outcomes, and contexts, until they were strongly supported with sufficient raw data from the identified papers. Themes within these constructs were developed iteratively and refined through a process of discussion and debate between the two authors. Once a final synthesized framework of themes and subthemes was established, codes were checked for consistency by KB and discussed and agreed with SJ. All the extracted data were appraised and considered but only indicative quotes and examples from papers rated as higher quality through the SURE quality appraisal process outlined previously are reported here as authors agreed that additional data would not have enhanced the theme.

#### Positionality

The review team consisted of clinical and academic professionals who brought complementary skills and expertise to the review process including developing the protocol, checking the sifting and screening process, and reviewing the reporting of results. KB and SJ are healthcare professionals who work clinically and research with people who use AAC and were responsible for analysing the data. KB and SJ both brought their experience of working in the field of AAC alongside their knowledge of the field of research to the review process. KB and SJ have different professional backgrounds (Speech and Language Therapist and Healthcare Scientist), which they used to check and challenge each other’s assumptions during the analytic process. KB and SJ were mindful that their clinical experience and concomitant assumptions risked obfuscating the perspectives presented in the papers reviewed. They responded reflexively and responsively to feedback from the wider team during data analysis, and worked collaboratively with the PPI group to rigorously scrutinize the generation of themes.

## Results

**Figure 1.**
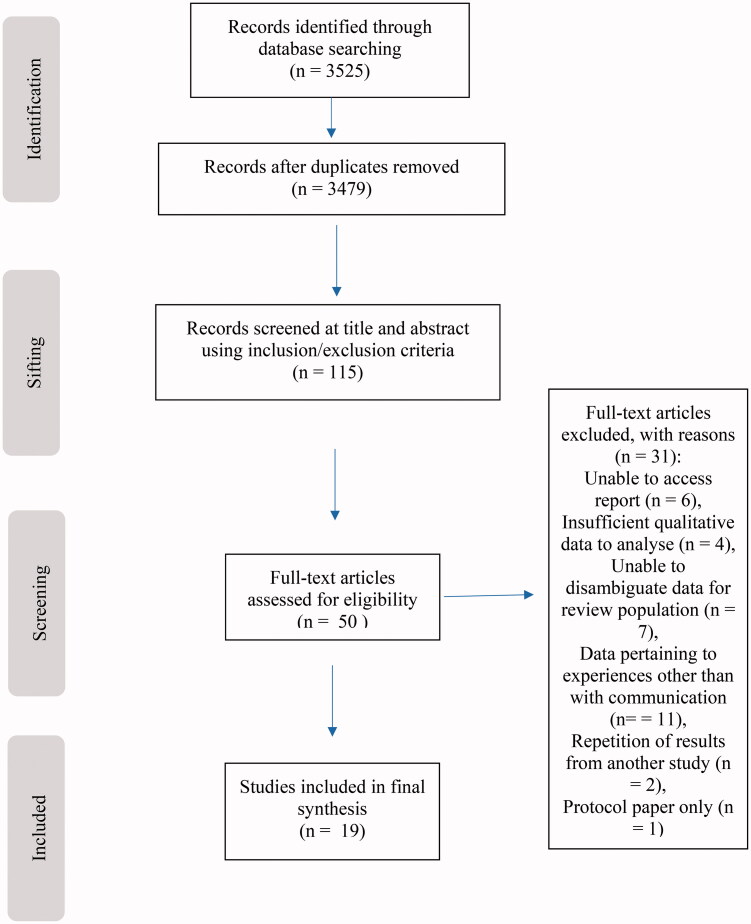
PRISMA flow diagram of screening process.

The initial search identified 3525 publications, resulting in 3289 papers of interest once duplicates were removed. Fifty papers were identified for further screening. Of these 50 papers, five were unavailable through the university library, document supply service or through attempting to contact the author directly. In one instance, translation services were unavailable. From the remaining 44 papers, 19 were included in the preliminary synthesis of the review (Figure 1).

Within the 19 papers informing the results of this review, the views of 204 participants were reported. People were represented with a range of congenital and acquired conditions such as cerebral palsy, Rett syndrome, stroke, traumatic brain injury, and amyotrophic lateral sclerosis. Papers represented research from six different countries: Australia, Canada, Ireland, Sweden, UK, and the USA (Table 1).

CERQual evidence profiles indicated that results could be viewed with a moderate degree of confidence. Overall, only minor concerns regarding the methodological limitations and adequacy of review reports were identified. All themes generated during the review were well-supported from data across studies of reasonable quality (Table 2).

**Table 2.** A summary of the CERQual evidence profile.

Summary of review findings	Assessment of review findings				Statement	
	Methodological	Coherence	Adequacy	Relevance	Confidence in Findings	Explanation
The value ascribed to communication aids is related to the effect that the device has on the individual’s sense of humanness. The outcomes from using AAC fall into two categories: communication and uses other than communication. Communication experiences are heavily influenced by contextual factors.	Minor limitations: Lack of description of researcher reflection or reflexivity during data collection or analysis. Lack of detail reported on the rationale for methodological choices and/or process.	Moderate concerns: Analysis tended to be descriptive rather than transformative. Limited representation of non-verbal communication in primary data. Little evidence of any contradictory data presented outside of a binary analysis e.g., ‘barriers/facilitators’	Minor concerns: Adequacy of the data richness and the number of participants was generally fairly good. Findings are supported from the data available in review papers.	Moderate concerns: In papers exploring notions beyond the scope of the review question, relevant data had to be extracted from within the primary data in response to the question.	Moderate	Minor concerns regarding the methodological limitations and adequacy were identified. The overall themes from the review were well-supported from data across most studies of reasonable quality within the review. In sub-themes less strongly represented in the data i.e. More than a Voice, data from high quality papers support themes however confidence in the evidence for these is less than for other findings.

**Figure 2.**
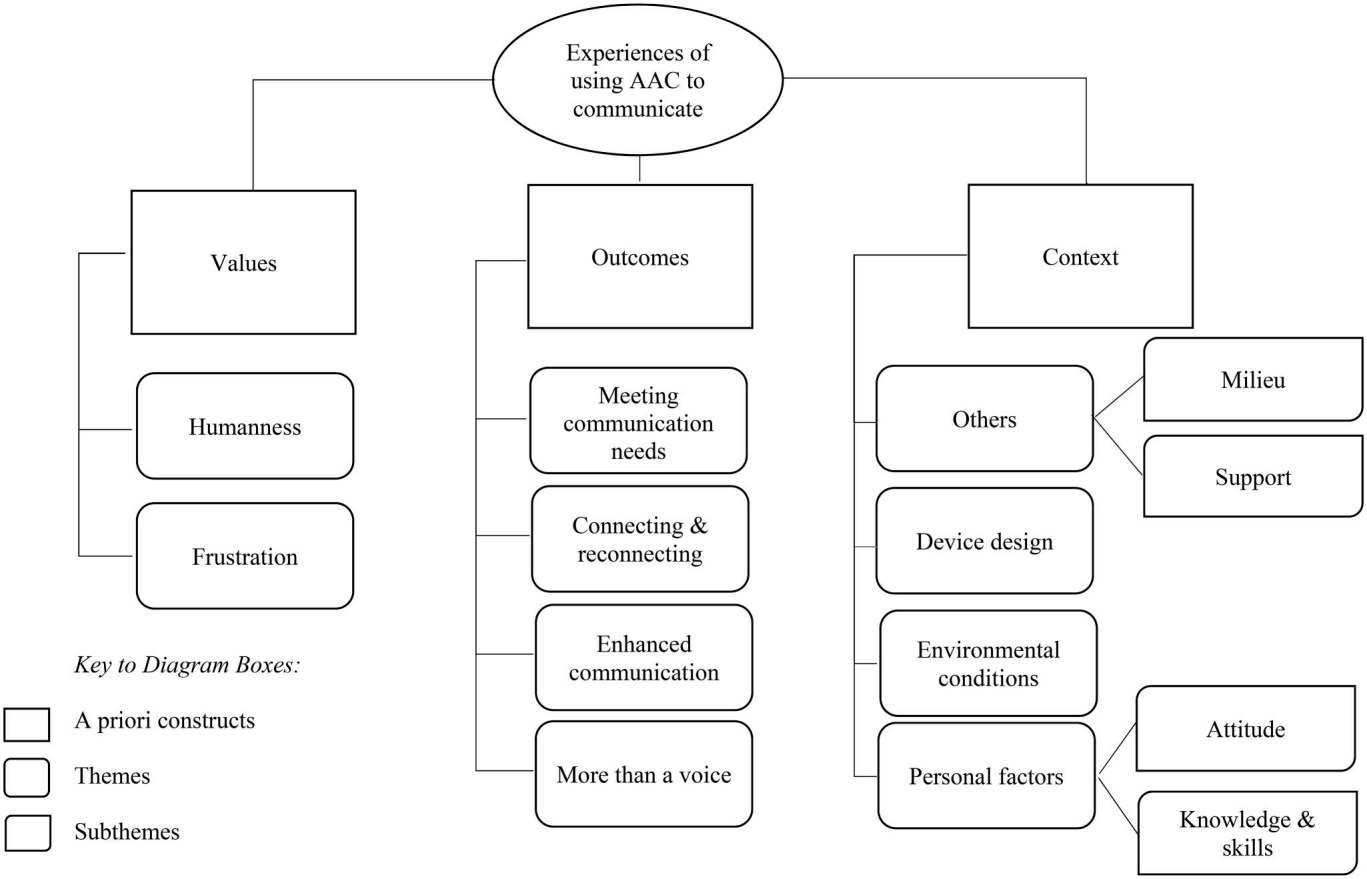
A map of the themes and subthemes generated during data analysis.

The communication experiences of people who use AAC were mapped onto three *a priori* constructs: values, outcomes, and contexts, with ten themes and subthemes generated during analytic analysis. Figure 2 sets out a map of the constructs themes and subthemes. A description of the themes and subthemes is presented below under the relevant construct heading with further supporting examples from the extracted data available in the supplementary material (Supplementary file 1).

### Values

This construct encapsulated ideas concerned with the emotional response to, or personal value associated with being able to communicate using AAC, and consequently the meaning that AAC holds for the individual. Two themes were generated that represented values: (a) the key to humanness and (b) frustration. Dickerson et al. [33] used the term “humanness” to capture the power and limitations of AAC in terms of an individual’s role, identity, and independence. AAC was, at times, the key to humanness but was also the cause of frustration and the route of feelings of alienation.

#### The key to humanness

AAC provided people with a greater sense of control and independence: “Geoff considered that he had more control over interactions with his family; e.g., ‘I don’t have to wait to be asked’” [34, p. 1527] and attributed this change to using high technology communication aids.

AAC provided people with an improved sense of self-identity and self-actualization:

“I am increasing my believing in myself. I can do everything I want to on my computer” [35, p. 94].

AAC also improved access to opportunity: "I needed an AAC device to get a job and, sure enough, as soon as I had my AAC device I was offered a job" [33, p. 218]. It enabled people to connect with a sense of normalness: “Particularly with people I don’t know very well I can come across as normal. Which is good…I think people generally make snap judgements about others based on their looks” [34,p.1527].

#### Frustration

Frustration frequently resulted in people who use AAC not being able to express themselves or their sense of humanness. For example, participants described a disconnect between their thoughts and their ability to express those thoughts:

I was in grade 7 when I got my first device. I was so excited. But as I got to school and tried to talk to my friends I just couldn’t get the words out fast enough. I am not a slow thinker, but even with my new device I am a slow talker. [36,p.44]

Participants were also frustrated that the computer voice of an AAC device could not express their personality sufficiently: “Personally I don’t like using it because using a computer system, there’s no personality there’s no, it’s very much factual… So I do shy away from the machine myself” [37,p.81].

People found the lack of privacy associated with using AAC frustrating: “The users ‘can't speak privately to a person over the telephone. And they can't speak to me without everybody hearing the conversation’” [33,p.218].

Participants experienced misinterpretation especially in text-based conversation on the internet: “It is very easy for people to misinterpret the meaning of email, instant messaging, or text messages, creating misunderstandings” [37,p.93].

### Outcomes

Outcomes are considered in this review as related to the purpose and product of using AAC. Most participants described the primary purpose of AAC as being related to communication at differing levels which are described in the following themes: (a) meeting communication needs, (b) connecting and reconnecting, and (c) enhanced communication. There were some data reflecting other uses of AAC devices concerned with functions other than communication per se that are summarized in the theme (d) more than a voice.

#### Meeting communication needs

There were several examples of participants using AAC to communicate basic, functional needs: “If I need something I can type it. Communication is a huge part of using this for me” [35,p.94].

Some participants relied on their AAC in specific situations such as using the telephone:

It helps me to talk to people on the phone. It is easy to use. I needed to get my lawn mower fixed. If I had not had this on my computer, they would not have understood what I wanted or who I wanted to talk to. [38,p.106]

Others described using AAC to participate in some form of specific communication task: “‘If you program your speech before [you do the presentation] you could just press a button and it would talk and it would do a lot of work for you’” [23,p.82], or to join in at school:

Sometimes though my teacher will give me a question that she’s going to ask the next day. And then I put my answer in my device that night so that when she asks me the next day I’ll be able to answer. [36,p.45]

For others AAC was a tool used to communicate in a range of settings:

The most common means of communication I use are speech through my AAC device, email, and instant messaging. I use AAC device speech throughout the day to communicate with my family and caregiver, both for normal conversation and to express needs I may have. [37,p.111]

#### Connecting or reconnecting

Participants described both making and maintaining relationships through facilitating conversation *via* AAC: "Voice output makes me part of the conversation by not needing someone to read my screen and relay it to others” [33,p.217]. AAC was also used to promote an individual’s inclusion:

I basically just log on, check e-mails, chat with people. So you are connected up with other people with disabilities and then those that don’t have disabilities. So it’s like a place where you can learn stuff from people. [35,p.93]

One participant developed mentoring relationships by supporting other laryngectomy patients to engage in AAC through using AAC:

I am on call at the hospital where I had my surgery to speak to any patient who would like to see and hear what a laryngectomy sounds like. I was speaking to a new laryngectomy; he was getting mad at trying to write, so I turned on my iPad and handed it to him. Within seconds, he was asking me all kinds of questions. I could see the look in his eyes that his wife was going to have to get him one. She has a smartphone, we downloaded the app and they started to really talk. [38,p.105]

AAC also opened up new opportunities to communicate with unfamiliar people and build new relationships: “I found love on a social media site and after some time and much courage we decided to meet [in person]… now we are happily married” [39,p.30].

#### Enhanced interactions

Participants described experiences that were about more than just getting a message across but creating a deeper connection and expressing more than a functional message: “If a question is complicated, you get a better response from the person by sending them an e-mail” [33,p.1525].

Participants also highlighted the importance of having access to multiple communication modes which provided flexibility and increased their chances of having successful interactions with new and unfamiliar communication partners:

INT: When you meet somebody for the first time in your volunteering work, do you use your speech or do you use your book or device?

Cathy: I use both sometimes. The book and the [SGD].

[23,p.80]

AAC enabled some participants to realize different or new roles in the workplace: “Carl noted that he preferred to use email to express his thoughts related to committee matters prior to meetings” [23,p.82].

Others described AAC as providing an enhanced mechanism for expressing detail about themselves to others: *“*[With social media] I have the opportunity to ACCURATELY represent myself to the world. The speed of communicating is non-existent” [39,p.30].

#### More than a voice

Several participants described tools, embedded within their AAC device, that facilitated them with other aspects of their day-to-day over and above communicating:

My AAC device is much more than my voice. It is how I access my computer, turn on/off things around the house; it is my voice and arms. It is even my memory as I have an address book, my schedule, checkbook and soon the bible. So I use it for a lot more than talking. [33,p.218)

School-age children used their AAC device to complete homework:

Well, it makes everything faster for me to do for homework and stuff. It’s easier for me and even my school work is faster to do, and my homework because sometimes I have to write essays and stuff and I use it to type. [35,p.94]

Whereas adults used their AAC device to help with completing work tasks: “Some participants commented on the flexibility of their communication device: “‘I like work on the PC’; ‘I can use it for several different things, email, internet work, etc.’; ‘looks like laptop’” [40,p.267].

Others used aspects of their AAC device as cognitive aids to facilitate organization; “the NECAS aid met specific needs, such as supporting memory through use of a picture-based shopping list […] or an organizational aid in the form of a calendar” [41,p.397].

Accessible computer-based AAC devices could enable adults who acquire communication difficulties to access additional internet functions:

I would use my smartphone for texting, quick weather updates and phone calls. Now I text through email. I have replaced the phone weather apps with specific websites and replaced calls with email and messaging. [37,p.97]

### Context

Communication experiences are heavily influenced by the environment in which an individual exists. There was significant representation in the papers identified in this review of data related to contextual factors that influenced people’s ability to achieve their desired outcomes. The PPI group reiterated the importance of these factors during a discussion about the initial descriptive themes, hence this additional construct was added, post-hoc, to the *a priori* framework that informed the analysis. The following themes were identified that represented context: (a) device design, (b) physical environment, (c) personal factors (which contains subthemes related to people’s attitude towards an AAC device and their skill at using it), and (d) the impact of others (with subthemes separating milieu and support).

#### Device design

Participants described issues relating to design features in terms of the flexibility, compatibility, usability, reliability, and acceptability: “When the iPad came on the scene it was a massive leap forward in communication skills and people could understand you much better and of course you have the various apps that come with it” [34,p.1525].

Portability and durability were considered important features of an AAC device as they were both described as being critical to an individual’s ability to function in a range of places. People described how electrical cables and leads trailed from AAC devices, restricting their ability to move around: “There were complaints that cumbersome wires tethered users like an ‘umbilical cord’" [33,p.218]. Battery life was also an important characteristic of an AAC device as it dictated where people could go and for how long: “Battery life was also important for heavy users to ensure that their units worked in any setting” [34,p.217]. Sensitively designed and selected AAC devices that took account of portability and durability were celebrated: “It can go from place to place without a problem. It’s easy to carry, easy to use, and can be understood by most. Very simple when in the hospital” [38, p. 106].

Participants described features that enhanced or limited the usability of an AAC device. Some people described specific software features that supported their communication: “[I liked] the word prediction … because then I don’t have to type out all the [complete] words, it will just pop up. It just made things faster. It [voice output] does the talking for me” [35,p.93].

Well-designed hardware also enabled easier access to AAC devices: “It’s helpful to be able to change the key size and the on-screen keyboard size” [35,p.92]. These features needed to be set-up and supported well for them to be usable to the person using them however: "a device not overloaded with complex and numerous features that require the user to fight his way through a forest of arcane trails to find a tree of interest" [33,p.218].

Reliability was another feature that hindered people’s ability to communicate effectively at all times: “Although my newest device is great, still, I can be in the middle of chatting when it stops working. I have to shut down the computer and reboot, which spoils the flow of a conversation” [42,pp.100–101].

Although having synthetic voice output supported communication, “The synthetic voice [iPad] is very easily understood” [33,p.1527], the limitations of existing speech output technology were also frequently noted, “I have come across children who need to use AAC, but will not do it because they hate their voice. Their aids are put away in cupboards” [42,p.102].

#### Environmental conditions

Data representing this theme were concerned with the compatibility between the AAC and the environment in which an AAC device was being used. Often AAC were adequate in certain conditions but not others. Participants discussed challenges concerned with the physical environment such as sunlight reflecting the screen of their AAC device or on eyeglasses: “Also I wear prescription glasses and the eye gaze [module] has trouble with reflection off of them on a sunny day. Consequently, if it is sunny out, I have to do my typing at night” [43,p.680]. Or, when in noisy environments, it was difficult to be heard: “There was a problem with background noise, and clarity was lacking. The biggest issue was lack of privacy” [38,p.106]. Some participants described a preference for certain types of AAC for use in specific locations: “I use sign language, the computer, a voice machine [AAC device] that I can use to talk on the phone and a TTY machine” [17,p.60].

#### Others

This theme represents the significance of the role of other people including friends, family, and society, and how they influence people’s experience of using AAC. Data were separated into two further subthemes: milieu and support.

##### Milieu

The term milieu is used to describe the social environment of the person who relies on AAC. The communication partner or listener is both prepared to listen to AAC-mediated communication and is an active listener, both of which were important to people who use AAC. Specific comments were made about how a communication partners’ attitude influenced the use of AAC: “Sometimes it was the partner’s attitude that influenced the use of strategies e.g., by preferring the informant to practise talking instead of using strategies” [44,p.150]. Another participant also observed that the attitude of the health professionals and caregivers changed when he was using his iPad: “They [staff] all act as if they were talking to me, not the iPad” [34,p.1525].

Several participants discussed the importance of others listening and acknowledging communication *via* AAC. Some discussed instances when partners were interested and helpful:

She always found the relay service operators to be “courteous, friendly, and helpful” and described both her role and that of the operator in tele- communication as being of equal importance: “It is a team effort. It’s my role to sufficiently assist the relay operator so that he or she can best assist me with the call.” [38,p.106]

Some participants found it disheartening when partners lacked the patience to give them time to construct messages or became bored when listening to the speech output: “At first my friends waited to hear what I had to say, but after a couple of sentences they lost interest and had moved on to something else” [36,p.44]. Whereas others found it easier to ignore this behaviour and focus more on positive reactions: “Wendy, for example, reported that she did not care what other people think, and that she had mostly positive reactions to her use of her Community Request Cards” [41,pp.397–398].

Social media provided a forum in which the milieu had less of an impact on people’s experience of communicating using AAC:

Participants discussed how they perceived many of their communication partners as more comfortable communicating with them on social media. Participants reported that people who were afraid to communicate in person were willing to get to know them through social media. [39,p.30]

##### Support

This subtheme includes data concerning the practical role of partners in setting up AAC and teaching or enabling its use. The importance of accessing the correct support was evident through the experience of people who were well-supported in the practicalities of setting up AAC: “I am lucky that I have sons that can help me set up different things on the computer since I am a little technically challenged” [37,p.109].

Participants also described how a lack of support affected their ability to engage with their AAC: “She preferred to use an electronic device, but often it was unavailable because the batteries were flat, although it became evident during the interview that her DSWs thought it was broken” [41,p.397].

The role of communication partners as advocates of AAC-use was considered important by participants and researchers. Family and teacher support was perceived as an enabler for use by the adolescent participants. “They [my parents and teachers] encouraged me not to give up” [35,p.92].

Participants struggled with a lack of training to use their AAC: “when asked by the researcher how much time the therapist spent explaining how the device worked, one of the participants responded, ‘Not much time spent with me’” [45,p.266].

#### Personal factors

This subtheme refers to data concerning the attitude towards AAC devices and the knowledge and skills of the individual who uses AAC in relation to their experience of communicating.

##### Attitude

Whether people had a positive or negative experience of using AAC was often influenced by their psychological outlook. Participants with a more negative attitude towards their AAC often chose not to engage with it: “Some felt silly using strategies because they perceived using them was an ‘abnormal’ means of communicating” [44,p.150]. Others felt like accepting AAC was akin to accepting defeat*:* “As I said to my speech and language therapist, it’s a nice toy but it’s like admitting defeat to not use my own voice” [45,p.264]. Others assumed a position of acceptance of AAC as an unavoidable compromise: ‘‘Obviously my [SGD] is slow, that makes the conversations slow, but I would prefer to have a communication device rather than having a communication board” [23,pp.80–81]. Whereas some participants were positive in their attitude towards AAC: “[communication aids] are essential” [34,p.1527].

##### Knowledge and skills

Participants described challenges associated with their physical abilities. For some people limited hand function impeded use of AAC:

Wife: So what do you feel about the Lightwriter^TM^ yourself?

Participant: Not much

Wife: It’s no very good because, if your fingers are stiff because of the keys, I think you feel by the time you’ve punched in an answer to some- body, the communication’s gone half a mile down the road [both laugh]

Wife: It’s too small and fiddly for you

Participant: Problem with my hands

[45,p.265]

Even participants who were able to directly access their AAC devices reported how reduced speed of movement affected the rate at which they could communicate: “I don’t like to get on live chat because I don’t type fast…so slow typing is my only barrier” [39,p.32].

Others described more general and fluctuating physical skills that affected how well they could engage with AAC: "One of the rare times when my device is useless is when I am mad and I want to get something out fast but to do that I have to relax and gain control" [33,p.217].

Participants described how limitations in their knowledge about using or programming their AAC device was a barrier to being able to engage with it: “Some participants commented on their own lack of knowledge, for example ‘do not know how to trouble shoot problems’ or ‘you are able to transfer text from a PC onto a Lightwriter^TM^, but I don’t know how to. This would be very handy’” [40,p.265]. Another reflected on how his own lack of skill limited his use “would like to be better at reading” [40,p.265].

## Discussion

This qualitative evidence synthesis identified data from existing research literature that provide greater insight into what people value about AAC and the outcomes that are important to them when using AAC. These data provide insight gained directly from the perspective of those who use AAC and specifically AAC devices. The findings of this QES establish that values can be framed in terms of the extent to which AAC enables humanness. Most participants, who were people who used AAC devices, described the primary purpose of using AAC as enabling communication, but this can be separated into communicating for different purposes: (a) meeting communication needs, (b) connecting and reconnecting, and (c) enhanced communication. Participants also described using their AAC for tasks unrelated to communication per se, such as planning and organization. Issues concerning the context in which an individual uses AAC were prevalent throughout the literature and were also discussed at length with the PPI group. It became clear during data analysis that the context in which they existed underpinned participants’ views on the extent to which AAC enabled both humanness and communication.

The findings from this QES can be used to inform a conceptual framework on which to build a PROM tool. PROMs can be used in practice by clinicians with those who use AAC and those considering use of AAC devices in reporting their experiences as well as understanding their goals. Further attention to the core constructs of values, outcomes and contexts will enhance our understanding of the underlying concepts of a PROM, and help to identify words and terms that have greater resonance for people who use AAC. Deeper, analytic synthesis of themes in existing qualitative data identified during this QES also enabled a more complex and nuanced appraisal of some of the issues concerned with qualitative research with people who use AAC. Employing QES methodology to address the research question enabled the research team to look at data across the heterogenous groups of people who use AAC and to identify the commonalities in their experiences of communication. This method also identified some challenges of conducting qualitative research concerning the perspectives of people who use AAC which will need to be attended to in future research projects.

### Words and meaning

One of the strengths of the findings from the current QES is that the themes generated echo those in previous reviews of a similar nature [2,19]. This consistency reinforces our understanding of the common experiences of using AAC such as being able to communicate basic needs, communication for building relationships, and using AAC to access further opportunities. Further attention to the overarching constructs of values, outcomes, and contexts and what these represent to people who use AAC would enhance a conceptual framework for a PROM. It is important to establish terminology that resonate with people who use AAC.

The words *values* and *outcomes* were seldom mentioned in the retrieved papers. These words were presented to, and their meaning was explored with, the PPI group who found them to be ambiguous and intangible. Existing frameworks used within AAC research provide helpful theory for clinical practice [13] but the terminology used within them may hold little meaning for people who use AAC. Researchers and clinicians are increasingly expected to involve patients and the public in research projects and developing services [28]. They therefore have a responsibility to improve the emphasis placed on co-constructing meaning and the effort afforded to establish a shared understanding of the words selected for use. PROMs can be useful patient-clinician communication tools [8] but only when they reflect concepts and language that have shared meaning. Terminology, and the meaning that words imply to the end-users, need to be carefully considered when developing a PROM or any other clinical tool for use with people who use AAC.

### The sphere of experience

There is tendency in qualitative research concerning AAC to describe results in terms of barriers and facilitators [16,17,22,34,40,45] whereas individual experience is rarely binary. This phenomenon was particularly prevalent within themes relating to context. Contextual factors that diminished some people’s experience of communicating using AAC enhanced the experience of others. For example, the attitudes of people around the person using AAC portrayed a broad spectrum of experience that was interpreted very differently; attitudes that proved to be barriers for some people who use AAC had little impact on others. One of the challenges of developing PROMs is accounting for response shift; that is being able to connect the intervention provided to a change in reported outcomes as distinct from other extraneous factors that could influence the individual’s perception of their altered status [50]. Gathering of additional, contextual information is one means suggested for understanding the phenomena of response shift and being able to better understand the data provided by PROM tools [50]. Perhaps then, what is more important than identifying general barriers and facilitators to the use of AAC is to understand the extent to which specific elements of an individual’s context or life story are of import to them at the point in time in which they interact with a clinician or researcher and how these may or may not influence outcomes from AAC interventions such as use of an AAC device.

Engaging the individual who requires AAC in dialogue concerning the degree to which factors within their context have an impact on their communication may provide a valuable additional perspective to clinical assessment and decision-making. It is important to note that what is significant to an individual on one day or in one situation, may not resonate as strongly on a different occasion. When developing clinical tools, such as PROMs, that reflect the nuance and dynamism of individual experience, researchers must listen carefully to people who use AAC; they must seek meaning beyond the words used during data collection interactions to construct a sympathetic understanding of the range of experiences that influence how, when, and why people interact with AAC, or not. Qualitative researchers working in this field need to consistently reflect and report on how their own experience and background influences their research in order that the transparency, rigour, and quality of their research is evident.

### Collaborative research partnerships

The PPI group who supported the research team with this review provided highly valuable insights on the process of conducting the QES. The group informed the evolution of the framework used to support the analytic stage of data synthesis by emphasising the importance of contextual aspects of the experience of using AAC that were present, but not strongly reported in the data extracted from the research papers. The comparative lack of supporting quotes for the theme ‘more than a voice’ despite its perceived importance to the PPI group may arise for several reasons. The search methods and inclusion criteria selected for this QES may have precluded relevant papers. It may be that the use of AAC devices for purposes other than communication is evolving faster than our ability to research how they are being used. Alternatively, there may be a delay between the flexible and creative ways in which people who use AAC use their devices and what clinicians and researchers are able to respond to. The results of this QES suggest that the use of AAC devices for more than communication is an area that warrants further investigation in future.

Reflecting on the literature identified by this QES will help to inform further areas and methods for research enquiry in future. Working alongside people who use AAC adds integrity to research and helps to ground it in lived experience. Their perspectives can help to expand the conceptual frameworks upon which we base research and is equally important in the process of PROM development.

### Limitations and future directions

This QES did not include the perspectives of young people under the age of 12 as the research team considered that their experiences were likely to be significantly different to those of adolescents and adults and may be more greatly influenced by issues relating to literacy and education. It also excludes the perspectives of people with ASC and social communication difficulties as the authors considered that the motivation for using AAC and the experiences of communication may also be influenced by factors specific to this population. Future research to establish whether these hypotheses are relevant or accurate would add to the current level of understanding about people’s experiences, expectations, and outcomes from using AAC to communicate.

Several methodological limitations were consistently identified within the papers included in this QES while carrying out the SURE checklist and completing the CERQual grading. Firstly, there was the lack of detail reported concerning the role of the researcher during data collection. Secondly, researchers did not reflect on their personal influence, potential biases, nor positionality during the process of data collection and analysis. Finally, data analysis was frequently descriptive rather than transformative and there was a general lack of depth in the analyses. The nature of co-constructed meaning that is a common feature of interactions with AAC was not well represented in the results reported in the studies identified. Data was presented in the form of direct quotes from participants who used AAC and did not attend to the role of the communication partner/interviewer in supporting interpretation.

Structural challenges to people who have communication difficulties, in physical and attitudinal barriers to disability, continue to pervade societies and are therefore likely to influence peoples experience of and the outcomes from using AAC. Much as the authors recognize these are additional, significant contextual and environmental factors, they were not identified in the data in this review. This may suggest that further research into the impact of these broader societal influences and the effect that they may have on the outcomes from AAC is warranted.

## Conclusion

This report presents a rich analysis of the qualitative literature concerning the values and outcomes that are important to people who use AAC and establishing the significance of each individual’s context on their experience of communicating using AAC. The findings can be used to inform the development of a conceptual framework for a PROM for AAC. The report enhances the existing literature concerning the experiences of people who use AAC by synthesising research from across the population regardless of underlying condition or type of AAC used. It focussed exclusively on papers reporting the perspective of the individual and was conducted with people who use AAC to generate a credible interpretation of the data.

The results of the QES, coupled with the impact of the PPI group on the research process, indicate that it is incumbent on researchers and clinicians to work alongside people who use AAC to develop products, terminology, and research that is meaningful, relevant, and authentically represents their experience.

We have no known conflict of interest to disclose.

## Supplementary Material

Supplementary_material.docx
