# Supplementary material for: A qualitative evidence synthesis of the experiences and perspectives of communicating using augmentative and alternative communication (AAC)
Source: Disabil Rehabil Assist Technol. 2022 Aug 26;19(5):1802–16. doi: 10.1080/17483107.2022.2105961 (PMC9612935; doi:10.1080/17483107.2022.2105961)
Supplement: Supplementary_material.docx [file IIDT_A_2105961_SM3257.docx]

Appendix 1

*A summary of further example quotes to support the themes and subthemes described in the results section*

| Theme | Subtheme 1 | Subtheme 2 | Example quote | Paper |
| --- | --- | --- | --- | --- |
| Values | The key to humanness |  | “Geoff considered that he had more control over interactions with his family; e.g. ‘I don’t have to wait to be asked’ and attributed this change to using high technology communication aids”  “I am increasing my believing in myself. I can do everything I want to on my computer”  "I needed an AAC device to get a job and, sure enough, as soon as I had my AAC device I was offered a job”  "My device lets me say what I want and be like a normal adult with my friends"  “I have feelings of independence when emailing get to express my feelings and thoughts to my kids and friends and family” | [33]  [34]  [32] |
|  | Frustration |  | “Talking to people feels a bit slow. It can be frustrating. I have a lot of thoughts in my head and it’s slow to do that”  “Personally, I don’t like using it because using a computer system, there’s no personality there’s no, it’s very much factual . . . So I do shy away from the machine myself...”  “The users ‘can't speak privately to a person over the telephone. And they can't speak to me without everybody hearing the conversation’” | [33]  [22]  [32] |
| Outcomes | Meeting communication needs |  | “If I need something I can type it. Communication is a huge part of using this for me”  Informant: You have to do like this . . . when you try . . . when you can’t speak so well.  "In all environments, we all still rely on gestures to a great extent"  "It helps me to talk to people on the phone. It is easy to use. I needed to get my lawn mower fixed. If I had not had this on my computer, they would not have understood what I wanted or who I wanted to talk to" | [34]  [43]  [32]  [37] |
|  | Connecting or reconnecting |  | "Voice output makes me part of the conversation by not needing someone to read my screen and relay it to others", "I use my communication device to talk to my wife and kids, they insist, they don't read my mind well"  “By using social media it also gives you the opportunity to make contact with new people from your friend's friends”  “I basically just log on, check e-mails, chat with people. So you are connected up with other people with disabilities and then those that don’t have dis- abilities. So it’s like a place where you can learn stuff from people”  “We had a family picnic during a summer holiday at my sister’s house. She has a waterfront home with a dock that had tables and chairs set up on it. I was able to sit there and participate in the conversation without holding it up. I was even telling jokes. It was one of the best times I have had since the operation” | [32]  [36]  [34]  [37] |
|  | Enhanced interactions |  | “If a question is complicated, you get a better response from the person by sending them an e-mail”  “[With social media] I have the opportunity to ACCURATELY represent myself to the world. The speed of communicating is non-existent”  "I would love to be able to express emotion as a speaker. I am able to grab the audience's attention because they are intrigued, but if I was able to express myself better with voice inflections it would make my speeches that much better and impacting"  INT: Are there challenges related to communication? Luke: ‘‘Not now because I’ve got [SGD].’’ INT: How important is having the [SGD] to your volunteering? Luke: ‘‘Very, very’* | [33]  [38]  [32]  [22] |
|  | More than a voice |  | “To sum it up, my device is a lot more than my voice. It is my hands, pencil and paper, my keyboard and mouse, my remote, my memory and even my best friend, somebody who is always there for me”  “Well, it makes everything faster for me to do for homework and stuff. It's easier for me and even my schoolwork is faster to do, and my home- work because sometimes I have to write essays and stuff and I use it to type”  “I use the calendar to. . . to. . .keep track of my activities. Because I have brain damage, I have to have everything written down, I can communicate now, but tomorrow, if it is not written down, I forget” | [32]  [34]  [40] |
| Context | Device design |  | “When the iPad came on the scene it was a massive leap forward in communication skills and people could understand you much better and of course you have the various apps that come with it”    “[I liked] the word prediction . . . because then I don’t have to type out all the [complete] words, it will just pop up. . . . It just made things faster. It [voice output] does the talking for me”  “Although my newest device is great, still, I can be in the middle of chatting when it stops working. I have to shut down the computer and reboot, which spoils the flow of a conversation”  "I would love to be able to express emotion as a speaker. I am able to grab the audience's attention because they are intrigued, but if I was able to express myself better with voice inflections it would make my speeches that much better and impacting." | [33]  [34]  [41]  [40] |
|  | Environment |  | “Also I wear prescription glasses and the eye gaze [module] has trouble with reflection off of them on a sunny day. Consequently, if it is sunny out, I have to do my typing at night”  “There was a problem with background noise, and clarity was lacking. The biggest issue was lack of privacy” | [42]    [37] |
|  | Others | Milieu | “They (staff) all act as if they were talking to me, not the iPad”  “She always found the relay service operators to be ‘courteous, friendly, and helpful’ and described both her role and that of the operator in tele- communication as being of equal importance: ‘It is a team effort. It’s my role to sufficiently assist the relay operator so that he or she can best assist me with the call’”  “At first my friends waited to hear what I had to say, but after a couple of sentences they lost interest and had moved on to something else”  “Wendy, for example, reported that she did not care what other people think, and that she had mostly positive reactions to her use of her Community Request Cards”  “Participants discussed how they perceived many of their communication partners as more comfortable communicating with them on social media. Participants reported that people who were afraid to communicate in person were willing to get to know them through social media” | [33]  [37]  [35]  [40]  [38] |
|  |  | Support | “I don’t decide [which aid to use], the Lightwriter is simply there when I get up and out of bed”  “I am lucky that I have sons that can help me set up different things on the computer since I am a little technically challenged!”    “They [my parents and teachers] encouraged me not to give up.”  “It was noticeable that the participants in this study had received very little input in terms of how to use their devices. For example, when asked by the researcher how much time the therapist spent explaining how the device worked, one of the participants responded, ‘Not much time spent with me’” | [33]  [36]  [34]  [44] |
|  | Personal | Attitude | “Some felt silly using strategies because they perceived using them was an ‘abnormal’ means of communicating”  ‘‘Obviously my [SGD] is slow, that makes the conversations slow, but I would prefer to have a communication device rather than having a communication board”*  ‘‘[communication aids] are essential” | [43]  [22]  [33] |
|  |  | Knowledge and skills | “I don’t like to get on live chat because I don’t type fast…so slow typing is my only barrier”  "One of the rare times when my device is useless is when I am mad and I want to get something out fast but to do that I have to relax and gain control"    “Some participants commented on their own lack of knowledge, for example ‘do not know how to trouble shoot problems’ or ‘you are able to transfer text from a PC onto a Lightwriter, but I don’t know how to. This would be very handy’. Another reflected on how his own lack of skill limited his use ‘would like to be better at reading’” | [38]  [32]  [39] |

*Note: SGD = speech generating device
